# A Therapeutic Insole Device for Postural Stability in Older People With Type 2 Diabetes. A Feasibility Study (SENSOLE Part I)

**DOI:** 10.3389/fmed.2019.00127

**Published:** 2019-06-27

**Authors:** Sophie C. Regueme, Charles Cowtan, Mohamed Y. Sedgelmaci, Mark Kelson, Joël Poustis, Leocadio Rodriguez-Mañas, Alan J. Sinclair, Benjamin Dallaudière, Isabelle Bourdel-Marchasson

**Affiliations:** ^1^CHU Bordeaux, Pôle de Gérontologie, Bordeaux, France; ^2^South East Wales Trials Unit, School of Medicine, Cardiff University, Cardiff, United Kingdom; ^3^Hexabio Sarl, R&D Department, Pessac, France; ^4^Division of Geriatrics, Hospital Universitario de Getafe, Getafe, Spain; ^5^Foundation for Diabetes Research in Older People, Diabetes Frail, Luton, United Kingdom; ^6^Department of Radiology, CHU Bordeaux, Bordeaux, France; ^7^University of Bordeaux, RMSB, UMR 5536, CNRS, Bordeaux, France; ^8^CNRS, RMSB, UMR 5536, Bordeaux University, Bordeaux, France

**Keywords:** diabetes mellitus, type 2, postural balance, vibrating insole, neuropathy

## Abstract

The application of a stochastic mechanical noise has been shown to improve plantar touch sensitivity in patients with diabetic neuropathy and balance control. The present work aimed to test the feasibility of a specially designed vibrating device on gait and posture in older patients with type 2 diabetes with special interest on potential side effect (sensation of needles or tingling, dizziness or falls) before further investigations. For this, gait and balance tests were performed in 29 older out and in-patients (mean age 84 years, Barthel index ≥ 60/100) immediately before and after a 19 min plantar vibrating sequence, as well as 15 min after. These tests included posturographic measurements under eyes closed and static conditions and clinical gait tests (Short Physical Performance Battery and Timed-Up and Go tests). The results showed that no side effect was measured immediately, 15 min and up to 30 days after the vibration sequence. Besides, postural and clinical gait tests showed global positive effects at immediate and 15 min follow-up. Further investigation are now necessary to determine whether a daily stimulation sequence for a given time would lead to long-term positive effects on daily living (NCT01654341; https://clinicaltrials.gov/ct2/show/NCT01654341).

## Introduction

The most common long-term diabetic complication is diabetic sensorimotor polyneuropathy, which is involved in the pathogenesis of diabetic foot (1). This complication is known to affect both sensory and motor nerves and causes, among others, a loss of sensory perception by increasing the sensory threshold of mechanoreceptors (2, 3). This leads to postural instability (4–8) and an increased risk of falling and dependency.

Previous studies have shown that the application of a mechanical noise may enhance afferent sensory input by making the stimulus perceptible at lower amplitude (7, 9, 10). This led to improved touch sensitivity of patients with diabetic neuropathy (7) and an enhanced balance control in diabetic and/or older patients (5, 8, 11). These findings are clinically important as impaired sensation may not only lead to serious secondary medical complications, but also to an inability to perform everyday tasks such as walking, and can lead to functional dependency. It is therefore of great interest to further investigate the effects of a vibrating insole device on gait and posture in older people with diabetes in order to prevent impairment of balance stability.

The above-mentioned studies compared patients' stability with and without concomitant vibrations of plantar surface and the tests were performed within experimental environments. The aim of the present study was to test the feasibility of specially designed vibrating insoles on gait and clinical walking test and potential post-effects in order to determine if this portable and autonomous device can be used safely by the patient before further investigation.

## Materials and Methods

### Participants

Patients were recruited in the geriatric departments of the University Hospital of Bordeaux (France) from June to November 2014. Eligible patients were aged 70 and older with type 2 diabetes diagnosed for at least 2 years. Patients were excluded if they scored lower than 60 points on the Barthel index, if they scored 0 point on the Short Physical Performance Battery test, if they were unwilling or unable to provide consent, if they had feet ulcerations or if they had a pace-maker.

The study was conducted according to the declaration of Helsinki (2008) and was approved by the Local Committee for Human Protection in Biomedical Research. All patients provided written informed consent. SENSOLE study is a substudy of the European project MID-Frail (NCT01654341).

### Trial Design

This was a non-randomized pilot study.

### Intervention

**Figure 1:**
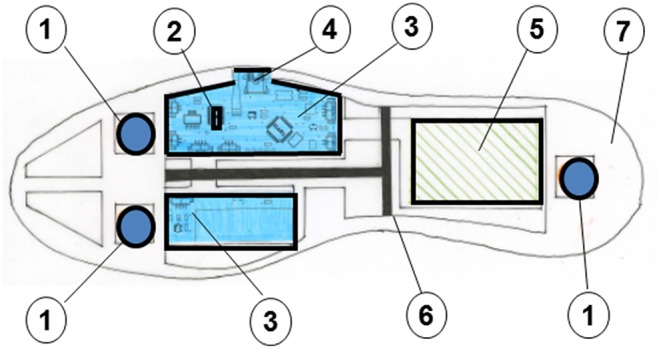
Details of the vibrating insole: **(1)** vibrating micro-motors; **(2)** microprocessor; **(3)** electronic device; **(4)** USB connector; **(5)** battery; **(6)** support sole; **(7)** superior membrane.

The vibrating insoles were prototypes specially designed by Hexabio (French Patents N° FR 2 951 566 [2011] and N° FR2 982 130 [2013]). As described in the Figure 1, vibrating micro-motors are settled in each insole under both anterior and posterior parts of the plantar foot surface that is near the first and fifth metatarsophalangeal joints, and under the heel. The membrane is also fixed in order to ensure a homogeneous vibrating noise under the whole forefoot and heel limiting the differences in foot characteristics among patients (e.g., focal callus, foot width, aging, and/or diabetes-induced foot deformation).

The motors are driven by custom-built micro-processor and the signal adjusted between 0 and 5 Volts delivers a random noise signal bandwidth varying from 0 to 200 Hz (maximal frequency of the device). The input for the three vibrating motors in a single insole is identical but the right and left insoles are independent so that the intensity of the vibration can be different under each of the two feet.

Prior to the vibrating sequence, individual threshold for perception was determined by increasing the vibration frequency from 0 to 200 Hz for each patient and each foot separately in a seated position. This consisted for the patient to notify the examiner once the vibration could be perceived.

Once the threshold for perception determined, the experimental vibration sequence was programmed at 90% of each individual's perception threshold in accordance with previous studies (5, 8, 11). The procedure consisted on two vibrating trains of 7 min with 5 min resting period in a seated position (total duration: 19 min). The vibration periods were interrupted for 5 min without vibration to avoid potential saturation of mechanoreceptors. The duration of the vibration periods was chosen so that the complete sequence lasted a reasonable time, facilitating a potential daily future use with a minimum of constraints for the patients. Throughout the procedure, the patient was in a sitting position, barefoot, each foot resting on the sole inserted in the platform. The sequence started with a remote control “on/off.”

### Outcome Measures

Severe neuropathy was tested using a 10 g Semmes-Weinstein monofilament in accordance with the guidelines of the International Working Group on the Diabetic Foot (12).

**Figure 2:**
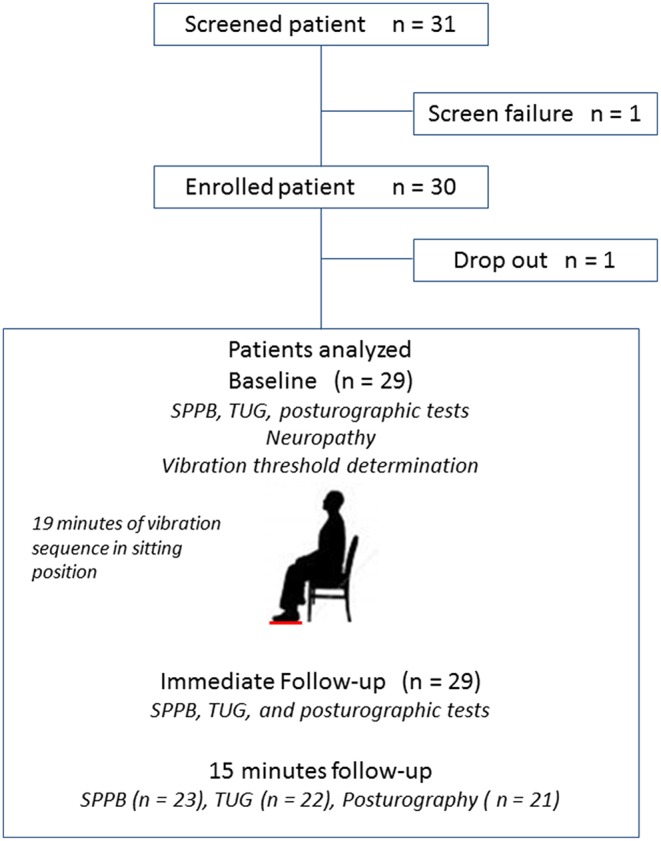
Study Flow diagram and design. Patient numbers at each stage of the study. SPPB, Short Physical Performance Battery; TUG, Timed Up and Go test.

Postural clinical tests were conducted immediately before (baseline) and after (immediate follow-up) the vibrating sequence, and were repeated 15 min after (15 min follow-up) (Figure 2).

The posturographic data were acquired by means of a double force platform (Feetest 6, Techno Concept, Mane, France). During the test, patients, stood with their feet abducted at 30 degrees and heels separated by 9 cm. In order to focus on potential plantar proprioceptive effects, the patients were required to close their eyes once their posture was stabilized. Two postural trials of 25.6 s were recorded with similar duration between trials. The recorded data included CoP sway area of 95% confidence ellipse, antero-posterior (AP) and medio-lateral (ML) CoP displacements, CoP velocity, and CoP velocity variance.

Clinical gait tests included the Short Physical Performance Battery (SPPB) and the Timed Up and Go tests. The SPPB test evaluates lower extremity functioning in older persons (13, 14) and includes balance, 4-meters walking, and repeated rising chair tests. The SPPB is scored between 0 and 12; with 0 indicating poor lower limb performance. The TUG test measures the time taken for a patient to stand up from an armchair, walk a distance of 3 meters, turn, walk back to the chair and sit down. The TUG test is a sensitive and specific measure for discriminating between fallers and non-fallers (15) with usual time about 6–12 s for community-dwelling older adults; the threshold for risk of falling being 20 s.

The potential side effect were recorded using adverse event reporting (e.g., sensation of needles or tingling, dizziness or falls) at any time during the study and up to 30 days after the vibrating sequence procedure.

Lastly, patient's age, gender, ambulatory status, medical history, and concomitant medications were also recorded.

### Sample Size

The sample size for this feasibility study was based on the ability to detect a mean difference in CoP sway area with sufficient precision that any harmful effect could be detected. With 30 patients (Figure 2) and assuming a standard deviation of 10.9^8^ a difference to within 5.516 mm^2^ either side using a 95% confidence interval was expected to be detected.

### Statistical Analyses

Complete case analyses of covariance (ANCOVA) was used for the CoP sway area change where follow-up CoP sway area scores are predicted using baseline CoP sway area and important patient characteristics [e.g., gender, age and baseline functional tests such as SPPB, TUG and the presence (or not) of severe neuropathy]. Logarithmic transformations to improve normality were also investigated if necessary. The comparisons of clinical gait tests (TUG and SPPB tests) between patients with and without severe neuropathy were realized using a repeated factor ANOVA analysis. *P* < 0.05 were considered to be statistically significant.

## Results

A total of 31 patients (15 male, 16 female) agreed to participate. One patient was not eligible based on his SPPB score (0/12) and one patient withdrew after screening. Therefore, data from 29 patients (mean age: 84.3 ± 6 years) were analyzed at baseline (Figure 2). Regarding gait and balance tests, all patients understood how to perform the tests, even if some patients required a demonstration.

The duration of assessments varied from 1 to 2 h depending on the autonomy of the patient. Some of them, with lowest Barthel scores, were unable to complete all the tests. Therefore, only 23, 22, and 21 patients completed, respectively, the SPPB, TUG, and posturographic assessments at the 15 min follow-up. Patient baseline characteristics, co-morbidities, and concomitant medications are presented in Table 1.

**Table 1:** Patients' baseline characteristics.

**Baseline characteristics (*N* = 29)**		
**MEASURE**		
**Gender**, ***N*** **(%)**		
Male	15	(52)
Age, mean ± SD	84.3	± 6
**Ambulatory status**, ***N*** **(%)**		
Cane	3	(10)
Two canes	2	(7)
Walker	2	(7)
Neuropathy, *N* (%)	8	(28)
Barthel score (/100), mean (SD)	90.5	(10.5)
**Co-morbidity based on MedDRA index**, ***N*** **(%)**		
Vascular disorders	28	(14)
Nervous system disorders	28	(14)
Cardiac disorders	21	(11)
Musculoskeletal and connective tissue disorders	18	(9)
Metabolism and nutrition disorders	16	(8)
Surgical and medical procedures	15	(8)
Renal and urinary disorders	14	(7)
Neoplasms benign, malignant and unspecified (incl. cysts and polyps)	9	(5)
**Concomitant Medication based on ATC index**, ***N*** **(%)**		
Alimentary tracts and metabolism	61	(31)
Nervous system	47	(24)
Cardiovascular system	45	(23)
Blood and blood forming organs	11	(6)
**Balance and gait scores at baseline (*N* = 29)**		
**CLINICAL TEST**		
SPPB, mean (SD)		
Balance test (/4)	2.7	(1.3)
Walking test (/4)	1.5	(1.5)
Chair test (/4)	1.0	(1.2)
Total (/12)	5.6	(2.8)
TUG (sec), mean ± SD	25.2	(20)
Normal (<10sec), N (%)	2	(7)
Good mobility (10-19.9sec), N (%)	14	(47)
Problems (> 20sec), N (%)	13	(43)
**POSTUROGRAPHY**		
**CoP sway area (mm^2^)**		
Patients without neuropathy, mean ± SD	438.2	± 438.6
Patients with neuropathy, mean ± SD	690.3	± 650.0
**CoP AP amplitude (mm)**		
Patients without neuropathy, mean ± SD	581.0	± 263.4
Patients with neuropathy, mean ± SD	684.0	± 379.2
**CoP ML amplitude (mm)**		
Patients without neuropathy, mean ± SD	255.0	± 122.7
Patients with neuropathy, mean ± SD	275.8	± 200.3
**CoP velocity (mm.s**^**−1**^**)**		
Patients without neuropathy, mean ± SD	26.2	± 11.9
Patients with neuropathy, mean ± SD	30.9	± 17.6
**CoP velocity variance (mm.s**^**−1**^**)**		
Patients without neuropathy, mean ± SD	499.1	± 423.4
Patients with neuropathy, mean ± SD	797.2	± 747.7
**Balance and gait scores at baseline (*N* = 29)**		
**Vibration threshold (Hz), mean ± SD**		
Patients without neuropathy	85.6	± 57.7
Patients with neuropathy	142.0	± 40.6

*Baseline characteristics, ambulatory status, co-morbidities and concomitant medications. SD, Standard Deviation; MedDRA, Medical Dictionary for Regulatory Activities; ATC, Anatomical Therapeutic Chemical classification system; SPPB, Short Physical Performance Battery; TUG, Timed Up and Go test; CoP, Center of Pressure; AP, antero-posterior; ML, mediolateral*.

Neither adverse reaction nor postural impairment was reported immediately after and 15 min after the vibration sequence for any patient. No adverse reaction was reported either within 30 days after the end of the follow-up period.

Twenty-nine of the 30 patients were included in the ANCOVA analysis of immediate follow-up which was controlled for baseline CoP sway area, SPPB, TUG, gender, age, and presence or not of severe neuropathy. Table 2 shows that for every 1% increase in CoP sway area at baseline, immediate follow-up CoP sway area increased by 0.82%. This implied that follow-up CoP sway area scores decreased by 18% after treatment.

**Table 2:** ANCOVA analyses at immediate and at 15-min follow-up, controlled for Baseline CoP sway area, SPPB total score, TUG, age, gender, and the presence (or not) of neuropathy.

	**Immediate follow-up**			**15-min follow-up**		
	**Coefficient**	**95% Lower CI**	**95% Upper CI**	**Coefficient**	**95% Lower CI**	**95% Upper CI**
CoP sway area^‡^	0.82	0.58	1.06	0.76	0.39	1.13
SPPB Total	0.05	−0.07	0.17	0.02	−0.16	0.20
TUG^‡^	0.07	−0.48	0.63	0.13	−1.08	1.34
Age	0.05	0.02	0.08	0.04	−0.02	1.00
Gender (Female)	−0.03	−0.41	0.36	0.04	−0.58	0.64
Neuropathy (Absent)	−0.11	0.19	−0.58	0.03	−0.59	0.64
CoP velocity	0.80	0.53	1.06	0.83	0.54	1.11
CoP variance^‡^	0.85	0.58	1.12	0.87	0.52	1.23
APCoPamplitude^‡^	0.93	0.66	1.21	1.02	0.58	1.45
MLCoPamplitude^‡^	0.86	0.51	1.21	0.78	0.51	1.04

*ANCOVA, ANalyse of COVAriance; CoP, Center of Pressure; AP, antero-posterior; ML, mediolateral; SPPB, Short Physical Performance Battery; TUG, Timed Up and Go test*.

‡ *Transformed using natural logarithm to improve model fit*.

Twenty-one of the 30 patients were included in the 15 min follow-up ANCOVA analysis, which was controlled for baseline CoP sway area, SPPB, TUG, gender and age. For every 1% increase in baseline CoP sway area, CoP sway area at 15 min follow-up increased by 0.76%. Again, this implied that 15 min follow-up CoP sway area scores were lower (by 24%) than baseline CoP sway area scores.

Of the non-transformed variables, both SPPB and CoP velocity reduced by 15%, for every 1 unit increase between baseline and immediate follow-up. A similar pattern of reduction was observed at 15 min follow-up for SPPB indicating a 21% reduction for every 1 unit increase at baseline. ML CoP amplitude showed the greatest reduction at 15 min follow-up, demonstrating a 0.78% increase for every 1% increase at baseline.

Of the transformed variables, CoP velocity variance showed the greatest reduction at immediate follow-up, demonstrating an increase of 0.85% for every 1% increase at baseline. TUG showed a minimal reduction at both immediate and 15 min follow-up, demonstrating an increase of 0.97 and 0.95% for every 1% increase of TUG at baseline.

Relatively to the repeated factor ANOVA analysis, the data showed global positive effects with a TUG time about 33.0, 32.5, and 29.8 m.s^−1^ at baseline, immediate follow-up and 15 min follow-up, respectively for the patients with severe neuropathy, and about 19.6, 18.8, and 16.1 m.s^−1^ for the patients without severe neuropathy. Similarly, the SPPB scores were 4.8, 5.5, and 4.8 for the patients with severe neuropathy, vs. 6.2, 6.5, 7.0 for the patients without severe neuropathy. None of these analyses was statistically significant.

## Discussion

The aim of this study was to assess the feasibility and the effects of specially designed vibrating insoles on both quiet standing and walking in older patients with type 2 diabetes in order to warrant further investigations.

The results showed that no adverse reaction such as sensation of needles or tingling, dizziness or falls were reported at any time during the study and up to 30 days after the end of the follow-up period. The use of device can be considered as safe as no impairment was reported neither in gait and postural tests immediately after and 15 min after the vibration sequence.

Although the amplitude of CoP displacement and sway area reported in the present study are consistent with the increased postural sway with aging on a static posturo-graphic platform, the present data were higher than previous studies in older people. Mean CoP sway areas varied for example from 100 to 400 mm^2^ in previous studies assessing faller and non-faller older patients in eyes-closed conditions (16, 17), compared with values of 500 mm^2^ in the present study. The mean TUG time value was also of 25.2 s with 43% of the patients taking over 20 s to complete the test, indicating a high risk of falling whereas previous studies report mean values of 8–9 s (16, 18, 19). The present TUG times were even higher than the mean times reported for older patients with intellectual deficiency (17.2 s) (20). These differences may be due to the heterogeneity of the patients' characteristics at baseline and to multimorbid study population, including older patients with severe neuropathy and instrumental and functional dependency. However, most variables were reduced by about 14 to 22% at immediate and 15 min follow-up, respectively. Looking specifically to the TUG and SPPB tests depending on the presence or not of severe neuropathy, the data showed a global positive effect of the vibrating insoles.

Despite some limitations of the present study (no control group, no long-term follow-up, no tuning fork to assess neuropathy at a less severe grade), this study demonstrated with aging an absence of adverse reaction related to the vibrating insoles. The design of this feasibility study did not allow to find similar results in previous studies (5, 8, 11). However; we observed a trend to positive effect on postural and gait assessment with sway area reduced by 18–24% after the vibration sequence, which is very encouraging. The next step will consist to a randomized-controlled trial with increased sample size to precise the efficacy of a daily use of these vibrating insoles on gait and posture.

## Data Availability

No datasets were generated or analyzed for this study.

## Ethics Statement

Ethics Committee: Comité de Protection des Personnes Sud-Ouest et Outre Mer III (30Nov2013). Regulatory autority (ANSM) 08Nov 2013.

## Author Contributions

Each of the authors has contributed to the conception and design or analyzes and interpretation of the data; has contributed to the draft or revision of the article, and has approved the final version of the article to be published.

## Contribution to the Field Statement

Diabetes in older patients is associated with increased frailty and functional decline. The application of a mechanical noise has been shown to improve touch sensitivity—in patients with diabetic neuropathy—and balance control. It is of great interest to explore plantar stimulation on gait and posture in older people with diabetes. The present manuscript builds on this knowledge and further investigates the feasibility and the safety of a home insole device for prevention of falls. We assessed here the potential side effects of specially designed vibrating insoles in 29 patients, aged 70 years or older, with type 2 diabetes and showed that o adverse reaction was reported during and after the procedures and that the sway area was reduced by 18–24% after the vibration sequence.

### Conflict of Interest Statement

The authors declare that the research was conducted in the absence of any commercial or financial relationships that could be construed as a potential conflict of interest.
